# Risk factors for subsidence of titanium mesh cage following single-level anterior cervical corpectomy and fusion

**DOI:** 10.1186/s12891-019-3036-8

**Published:** 2020-01-14

**Authors:** Chengyue Ji, Shunzhi Yu, Ning Yan, Jiaxing Wang, Fang Hou, Tiesheng Hou, Weihua Cai

**Affiliations:** 1 Department of Orthopedics, Shanghai Tenth People’s Hospital, Tongji University School of Medicine, 301 Yanchang Road, Shanghai, China; 2 Department of Orthopedics, the First Affiliated Hospital of Nanjing Medical University, 300 Guangzhou Road, Nanjing, Jiangsu Province China

**Keywords:** Anterior cervical corpectomy and fusion, Titanium mesh cage, Risk factors, Subsidence

## Abstract

**Background:**

To clarify the risk factors for subsidence of titanium mesh cage (TMC) following single-level anterior cervical corpectomy and fusion (ACCF) to reduce subsidence.

**Methods:**

The present retrospective cohort study included 73 consecutive patients who underwent single-level ACCF. Patients were divided into subsidence (*n* = 31) and non-subsidence groups (*n* = 42). Medical records and radiological parameters such as age, sex, operation level, segmental angle (SA), cervical sagittal angle (CSA), height of anterior (HAE) and posterior endplate (HPE), ratio of anterior (RAE) and posterior endplate (RPE), the alignment of TMC, the global cervical Hounsfield Units (HU) were analyzed. Clinical results were evaluated using the Japanese Orthopedic Association (JOA) scoring system and the Visual Analog Scale (VAS).

**Results:**

Subsidence occurred in 31 of 73 (42.5%) patients. Comparison between the groups showed significant differences in the value of RAE, the alignment of TMC and the global cervical HU value (*p* < 0.001, *p* = 0.002, *p* < 0.001). In multivariate logistic regression analysis, RAE > 1.18 (OR = 6.116, 95%CI = 1.613–23.192, *p* = 0.008), alignment of TMC > 3° (OR = 5.355, 95%CI = 1.474–19.454, *p* = 0.011) and the global cervical HU value< 333 (OR = 11.238, 95%CI = 2.844–44.413, *p* = 0.001) were independently associated with subsidence. Linear regression analysis revealed that RAE is significantly positive related to the extent of subsidence (r = − 0.502, *p* = 0.006).

**Conclusion:**

Our findings suggest that the value of RAE more than 1.18, alignment of TMC and poor bone mineral density are the risk factors for subsidence. TMC subsidence does not negatively affect the clinical outcomes after operation. Avoiding over expansion of intervertebral height, optimizing placing of TMC and initiation of anti-osteoporosis treatments 6 months prior to surgery might help surgeons to reduce subsidence after ACCF.

## Background

Anterior cervical corpectomy and fusion (ACCF) has been a highly effective treatment for various pathologies including posterior osteophytes of vertebral, ossified posterior longitudinal ligament (OPLL), prolapse of free nucleus pulposus, and tumors. It has been particularly shown to improve the long-term outcomes of patients as it sufficiently removes spinal cord compressions [1, 2]. After corpectomy, reconstruction of the affected area is achieved by implantation of autografts, allografts and bone substitute. Traditional autologous bone grafts are associated with donor site morbidities whereas allografts and bone substitute have been questioned due to high rates of pseudarthrosis and delayed union [3–5]. Titanium mesh cage (TMC) filled with autografts are widely used owing to their satisfactory clinical outcomes. The TMC shows good biocompatibility, and rapidly stabilizes the affected segments without causing bone graft-site morbidities [6]. However, TMC subsidence is frequently observed in the early postoperative period [7]. In current literature, 3 mm is chosen as the cut-off to separate subsidence from non-subsidence cases [8, 9]. Severe subsidence is correlated with poor neurological recovery and clinical outcomes [10]. Moreover, excessive subsidence into the adjacent vertebral bodies may cause instability, reconstruction failure and recurrence of neurological symptoms [11]. Therefore, effective interventions should be developed to control or even prevent subsidence. Several reports indicate that age, sex, endplate preparation and level of corpectomy are associated with subsidence but there are no known definitive risk factors for this phenomenon. This study aimed to identify specific risk factors for TMC subsidence to facilitate better management of this condition.

## Methods

### Study population

The present retrospective cohort study included 73 consecutive patients who underwent single-level anterior cervical corpectomy and fusion with the use of TMC (DePuy Spine, New Brunswick, New Jersey) between March 2012 and October 2015. To achieve stability, anterior plate (DePuy Spine, New Brunswick, New Jersey) was also employed in all the patients. Written and informed consent has been obtained from each patient. Corpectomies were performed for various pathologies such as posterior osteophytes of vertebral, OPLL and prolapse of free nucleus pulposus. There were 58 patients with cervical spondylotic myelopathy and 15 patients with OPLL. The enrolled patients included 37 males and 36 females and the mean age at surgery was 58.18 years (range 30–77). The level of corpectomy and fusion was as follows: C3 in 1 case, C4 in 14 cases, C5 in 29 cases and C6 in 29 cases. The exclusion criteria were: trauma, infection, tumor, posterior fusion, revision surgery and inability to measure due to the overlap of shoulders. Fifteen patients had one comorbid illness whereas 10 patients had more than one comorbidity. Hypertension was diagnosed in 16 patients, diabetes mellitus in 13 patients, and coronary disease in 6 patients.

### Surgical procedure

All operations were performed by an experienced spine surgeon (H. T.). Under general anesthesia, the patient was maintained in the supine position with neck slightly extended. We approached the cervical spine through a standard channel technique in all patients. Once the vertebral levels were carefully identified by intraoperative radiography, the intervertebral space was expanded with a distractor system. Following necessary discectomies, the vertebral bodies were removed and autologous bone chips obtained from corpectomy were used as bone graft material. Then remove the posterior longitudinal ligament and osteophytes; and carefully prepare the endplate in each case to minimize subsidence. After complete decompression, an appropriate length of TMC was selected and filled with autologous bone fragments taken from the excised vertebra, with end caps employed in some cases. The TMC was then inserted into the corpectomy defect and the location of the cage was confirmed using intraoperative fluoroscopy. Finally, an appropriately sized anterior cervical locking plate system was applied in all patients for further stabilization. The patients were instructed to wear Philadelphia collars for 6 weeks after surgery.

### Radiological assessment

**Fig. 1:**
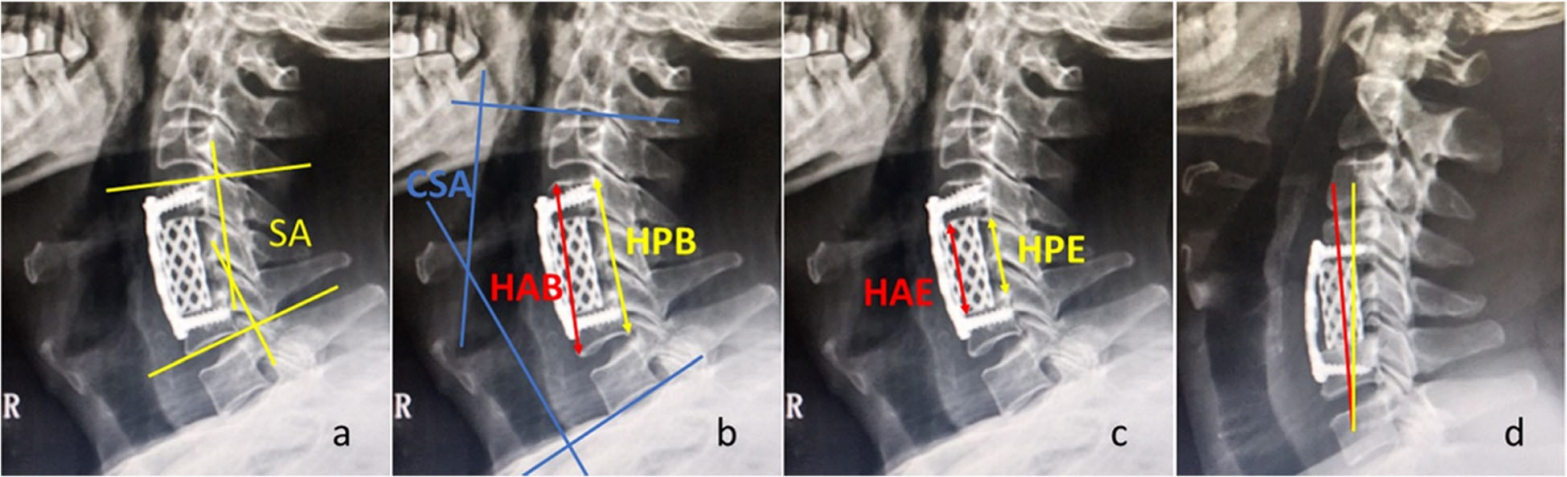
Sagittal radiograph showing a the segmental angle (SA) between the borders of endplates above and below the affected segment. **b** the cervical sagittal angle (CSA) formed between the lower endplate of C2 and C7. The height of anterior (HAB) and posterior border (HPB) were measured as the distance between the anterior and posterior points of the upper endplate of the superior vertebra and the lower endplate of the inferior vertebra. **c** The height of anterior (HAE) and posterior endplate (HPE) were measured as the distance between the anterior and posterior points of the lower endplate of the superior vertebra and the upper endplate of the inferior vertebra. **d** The alignment of TMC was defined as the angle formed between the central axis of TMC and the line through the midpoints of both endplates of adjacent vertebrae

**Fig. 2:**
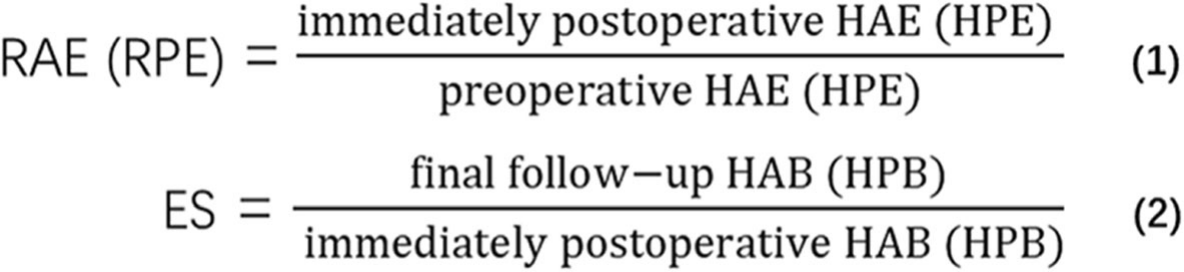
Formulas for calculating (1) ratio of anterior endplate (RAE), ratio of posterior endplate (RPE) and (2) extent of subsidence (ES). HAE, height of anterior endplate; HPE, height of posterior endplate; HAB, height of anterior border; HPB, height of posterior border

**Fig. 3:**
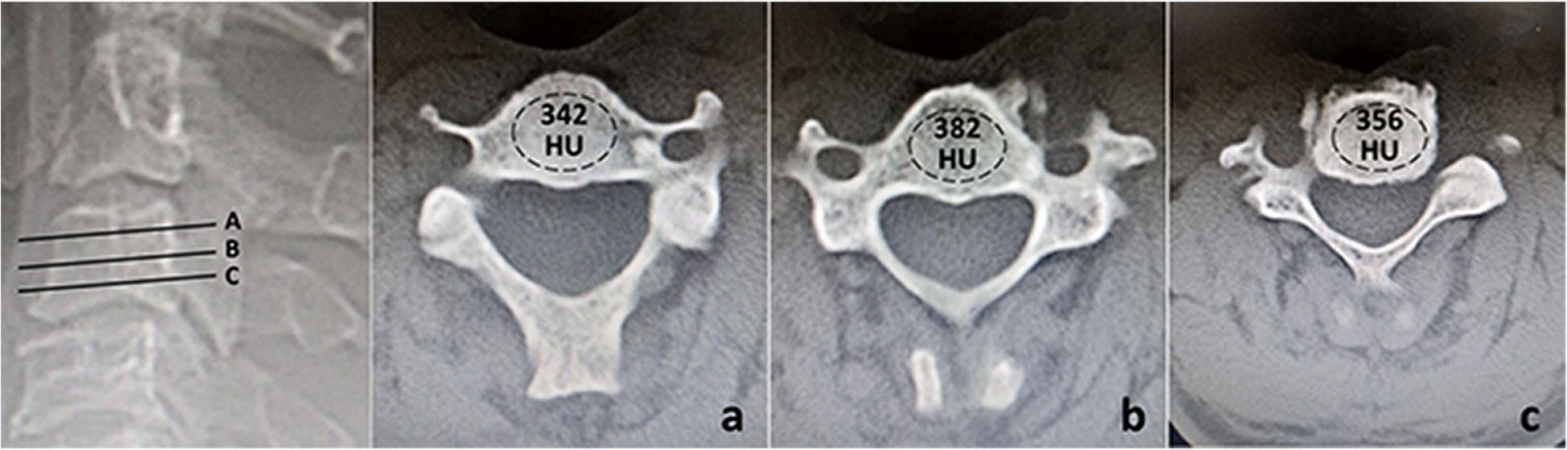
Using standard picture archiving and communication system (PACS), an elliptic region of interest (ROI) encapsulating only cancellous bone was drawn on three non-sequential axial images: **a** just inferior to the superior endplate, **b** mid-body, and **c** just superior to the inferior endplate. The software automatically calculated the mean Hounsfield Units (HU) value of each ROI. The average of HU values on 3 slices was regarded as the HU for individual vertebral body

Anteroposterior, lateral radiographs taken with the patient in a standing position were obtained before operation, immediately after operation. In addition, anteroposterior, lateral, flexion–extension lateral radiographs were taken at 3, 6, 12, 18, 24 months postoperatively and then annually thereafter. All the patients were observed for at least 24 months after surgery. Radiological assessment included segmental angle (SA), cervical sagittal angle (CSA), height of anterior (HAB) and posterior border (HPB), height of anterior (HAE) and posterior endplate (HPE), ratio of anterior (RAE) and posterior endplate (RPE), the alignment of TMC, the extent of subsidence (ES) and the global cervical Hounsfield Units (HU) value. The SA was defined as the angle between the borders of endplates above and below the affected segment (Fig. 1a). The CSA was defined by the Cobb angle formed between the lower endplate of C2 and C7 (Fig. 1b). The HAB and HPB were measured as the distance between the anterior and posterior points of the upper endplate of the superior vertebra and the lower endplate of the inferior vertebra (Fig. 1b). The HAE and HPE were measured as the distance between the anterior and posterior points of the lower endplate of the superior vertebra and the upper endplate of the inferior vertebra (Fig. 1c). The RAE (RPE) was the ratio of immediately postoperative HAE (HPE) to preoperative HAE (HPE), representing the extent of intervertebral space expansion during operation (Fig. 2). The alignment of TMC was defined as the angle formed between the central axis of TMC and the line through the midpoints of both endplates of adjacent vertebrae (Fig. 1d). Subsidence was defined as loss of more than 3 mm in any of the HAB and HPB measured heights compared with that immediately after operation. ES was the ratio of HAB (HPB) at the final follow-up to the HAB (HPB) immediately after operation, representing the extent of subsidence (Fig. 2). HU values obtained from CT scanning have been reported to be firmly correlated with bone mineral density (BMD) and we used technique described by Schreiber to measure the global cervical HU value [12, 13]. Using standard picture archiving and communication system (PACS), an elliptic region of interest (ROI) encapsulating only cancellous bone was drawn on three non-sequential axial images: just inferior to the superior endplate, mid-body, and just superior to the inferior endplate. The software automatically calculated the mean HU value of each ROI and the average of the 3 measurements was regarded as the HU for individual vertebral body (Fig. 3). The global cervical HU value was defined as the mean HU value of each vertebral body from C2 to C7. Bone fusion was judged by the absence of motion more than 2° between the spinous processes on flexion–extension lateral radiographs, the absence of radiolucent gap between the graft and end plate, and the presence of continuous bridging bony trabeculae at the graft-endplate interface. If there was doubt about the satisfaction of criteria on X-ray and the patient was symptomatic, sagittal reconstructed CT was performed for the judgement of fusion. In our research, 42 CTs were performed to examine the bone fusion. Movement of ≥2° on flexion/extension radiographs was regarded as a pseudarthrosis. These parameters were observed by a single spine surgeon who was not involved in the surgery.

### Clinical assessment

Clinical results were evaluated using the Japanese Orthopedic Association (JOA) scoring system for functional neurological status and the Visual Analog Scale (VAS) for arm and neck pain before operation, immediately after operation and at the final follow-up. All surgery-related complications including wound infection, dysphonia, dysphagia and implant-related complications were recorded immediately after operation, 3, 6, 12, 18, 24 months postoperatively and then annually thereafter.

### Statistical analysis

Statistical evaluation was performed using SPSS software. Results were presented as the mean ± standard deviation. Variables between the subsidence and non-subsidence group were compared using the Student’s t-test, Mann-Whitney U test, χ^2^ test and Fisher’s exact test. Factors with *p* < 0.05 were selected into the multivariate logistic model to identify the risk factors for subsidence of TMC. The best cut-offs of parameters were established using the receiver operating characteristics (ROC) curve. A linear regression analysis was performed to evaluate the relationship between the RAE and the extent of subsidence. The *p* value < 0.05 was considered statistically significant.

## Results

### Radiological outcomes

No patients were lost to follow-up in this study. All the patients were diagnosed and divided into subsidence group (31 patients, 42.5%) and non-subsidence group (42 patients,57.5%). In the subsidence group, the preoperative HAB and HPB were 54.56 ± 4.81 and 53.81 ± 4.93 mm. Immediately after operation, the HAB and HPB increased to 58.70 ± 4.70 and 56.24 ± 4.60 mm, respectively. At the last follow-up, these values were 54.13 ± 4.42 and 52.92 ± 4.57 mm. A significant difference was observed between the loss of anterior and posterior intervertebral height in the subsidence group(*p* = 0.001), indicating that the loss of anterior intervertebral height was more significant than that of posterior intervertebral height.

Comparison between the subsidence and non-subsidence group (Table 1) showed that there were no statistically significant differences in the CSA and SA. However, the value of RAE of the patients who experienced subsidence was 1.24 ± 0.08, which was significantly (*p* < 0.001) larger than that of patients in the non-subsidence group (1.12 ± 0.08). The alignment of TMC and the global cervical HU value were also found significantly associated with the risk of subsidence (*p* = 0.002, *p* < 0.001). The fusion rate in the subsidence and non-subsidence group was 27 patients (87.1%) and 39 patients (92.9%) 6 months after the surgery, respectively (*p* = 0.448). Bone fusion was achieved in all patients at the last follow-up.

**Table 1:** Comparison of the Subsidence and Non-subsidence groups

Factor	Subsidence Group (*N* = 31)	Non-subsidence Group (*N* = 42)	*P* value
Age(years)	59.39 ± 8.49	57.29 ± 12.85	0.708
Sex (male: female)	15:16	22:20	0.736
Operation level			
C3	0	1	
C4	8	6	
C5	11	18	
C6	12	17	0.660
Using end caps			
yes	19	18	0.119
no	12	24	
Preoperative CSA	14.13 ± 9.19	15.37 ± 10.47	0.675
Preoperative SA	6.94 ± 5.51	6.91 ± 5.37	0.995
Postoperative CSA	17.74 ± 9.71	17.80 ± 9.97	0.980
Postoperative SA	11.76 ± 5.83	11.18 ± 6.71	0.705
Change in CSA	3.61 ± 8.27	2.42 ± 9.44	0.588
Change in SA	4.81 ± 5.93	4.28 ± 6.60	0.727
RAE^*^	1.24 ± 0.08	1.12 ± 0.08	< 0.001
RPE	1.15 ± 0.07	1.11 ± 0.11	0.112
Alignment of TMC^*^	2.377 ± 1.9035	3.922 ± 2.0667	0.002
Global cervical HU value^*^	315 ± 73	388 ± 64	< 0.001

### Clinical outcomes

**Fig. 4:**
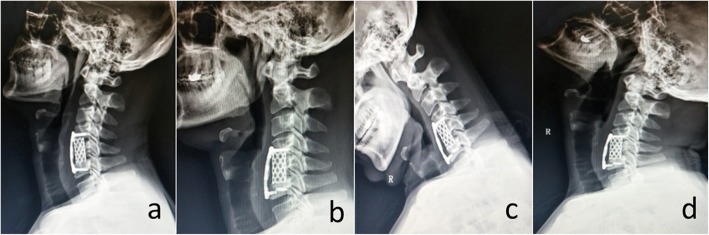
**a** A 43-year-old male patient underwent C5 corpectomy and fusion. **b** Lateral radiograph showed screw breakage while the subsidence was not obvious 1 month after surgery. **c, d** Flexion–extension lateral radiographs demonstrated poor bone fusion which may result to reconstruction failure

The JOA and VAS scores for arm and neck significantly improved in both groups immediately after operation and at the final follow-up (*P* < 0.001). However, there were no significant differences of the VAS and JOA scores between the subsidence and non-subsidence groups immediately after operation and at the final follow-up (Tables 2, 3). Moreover, no significant differences were observed in VAS and JOA scores immediately after operation and at the final follow-up in the subsidence group (*p* = 0.393, *p* = 0.385, *p* = 0.579). No patients showed wound infection, dysphonia after surgery. Dysphagia occurred in 3 patients (4.1%), and spontaneously resolved within 3 months postoperatively. Three patients underwent revision surgery for reconstruction failures (Fig. 4).

**Table 2:** Evaluation of VAS outcome

	Neck VAS			Arm VAS		
	Preoperative	Postoperative	Final follow-up	Preoperative	Postoperative	Final follow-up
Subsidence Group	7.10 ± 1.07	2.55 ± 1.06^*^	2.84 ± 1.15^*^	7.16 ± 1.15	2.77 ± 0.95^*^	2.97 ± 0.91^*^
Non-subsidence Group	7.14 ± 1.18	2.64 ± 1.12^*^	2.90 ± 1.30^*^	6.79 ± 1.13	2.60 ± 1.01^*^	2.69 ± 1.29^*^
*P* value	0.807	0.772	0.805	0.194	0.286	0.273

* *p* <0.05

**Table 3:** Evaluation of JOA outcome

JOA			
	preoperative	Postoperative	Final follow-up
Subsidence Group	8.39 ± 1.56	13.03 ± 1.14^*^	12.84 ± 1.29^*^
Non-subsidence Group	8.17 ± 1.43	13.14 ± 1.04^*^	12.71 ± 1.01^*^
*P* value	0.544	0.632	0.728

* *p* <0.05

### Risk factors of subsidence

**Fig. 5:**
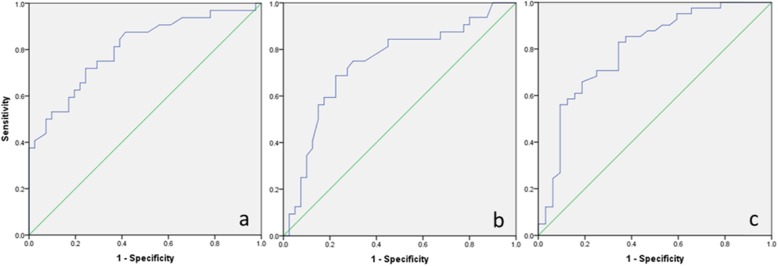
The ROC curves demonstrated that the areas under the curve (AUC) for **a** RAE, **b** alignment of TMC and **c** global cervical HU value were 0.803 (*p* < 0.001), 0.739 (*p* = 0.001) and 0.795 (*p* < 0.001)

The ROC curves (Fig. 5) demonstrated that the areas under the curve (AUC) for RAE, alignment of TMC, global cervical HU value were 0.803 (*p* < 0.001), 0.739 (*p* = 0.001), 0.795 (*p* < 0.001) and the best thresholds for RAE, alignment of TMC, global cervical HU value were 1.18 (sensitivity:71.9%; specificity:75.6%), 3 (sensitivity:68.8%; specificity:77.5%) and 333 (sensitivity:82.9%; specificity:65.6%). In multivariate logistic regression analysis, RAE > 1.18 (OR = 6.116, 95%CI = 1.613–23.192, *p* = 0.008), alignment of TMC > 3° (OR = 5.355, 95%CI = 1.474–19.454, *p* = 0.011) and the global cervical HU value< 333 (OR = 11.238, 95%CI = 2.844–44.413, *p* = 0.001) were independently associated with subsidence (Table 4).

**Table 4:** Multivariate Logistic Regression

Factor	Odds Ratio (95% CI)	*P* value
RAE > 1.18	6.116(1.613–23.192)	*P* = 0.008
Alignment of TMC > 3°	5.355(1.474–19.454)	*P* = 0.011
Global cervical HU value< 333	11.238(2.844–44.413)	*P* = 0.001

**Fig. 6:**
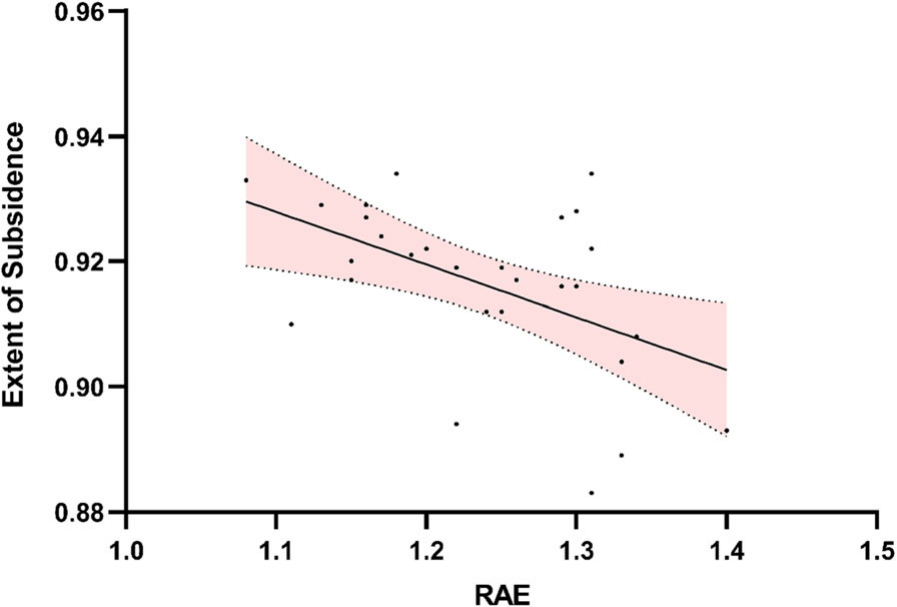
Regression plots showing the relationship between the ratio of anterior endplate (RAE) and the extent of subsidence (ES). Note the upper and lower boundaries of the 95% confidence interval

Moreover, the value of RAE had a significant effect on the extent of subsidence. As evidenced by the simple linear regression (Fig. 6), the subsidence of TMC became more serious with the increase of RAE (r = − 0.502, *p* = 0.006).

## Discussion

Anterior cervical decompression and fusion is one of the efficient methods of treating degenerative cervical diseases with satisfactory results. When the compression is extended to the vertebral body levels, anterior cervical corpectomy and fusion (ACCF) offers sufficient operating space and decompression extents [14]. However, subsidence of TMC was a common phenomenon after surgery. According to previous studies, subsidence of TMC range from 9 to 79.7% because of different definitions [7, 15, 16]. In our study, subsidence rate was 43.5%. The loss of anterior intervertebral height due to subsidence was more significant than that of posterior intervertebral height, which was different from previous studies [17, 18].

Some studies have shown that many factors may affect subsidence. It is generally accepted that the risk of subsidence may increase in elderly people and in women because of the osteoporosis associated with the ageing process [19]. However, differences in age and sex did not reach statistical significance in our study. Wu et al. [17] noticed that the C6 corpectomy was more susceptive to subsidence due to the gradient of adjacent endplates which made the contact surface too small and increased the compressive force on the endplates. Our study indicated that the level of corpectomy did not significantly affect the subsidence of TMC, which was consistent with some previous literature [20, 21]. In order to increase contact area, end caps were employed to decrease the incidence of subsidence. Nevertheless, we found that use of end caps did not reduce the risk of subsidence as expected. Cervical sagittal angle and segmental angle had also been underlined in previous literature [22] while our study found that there were no statistically significant differences of these factors between subsidence and non-subsidence groups.

In the present study, the risk of subsidence might increase in patients with over expansion of intervertebral space during operation, which was represented by the value of RAE. In a biomechanical study, increased higher distractive force on the endplate was recorded with lager graft. Furthermore, the distractive force required for graft insertion and subsequent graft compressive force were linearly related [23]. Moreover, Linear regression analysis revealed that RAE is significantly positive related to the extent of subsidence. Comparing with decreased value of intervertebral height, we believed that the ratio of final follow-up intervertebral height to immediately postoperative intervertebral height could truly reflect the extent of the subsidence due to the individual differences. Over expansion of the anterior border exactly explained the characteristic of subsidence in our study. Although the restoration of the intervertebral height has been considered as the key point to achieve satisfactory clinical outcomes, this result revealed that surgeons should be vigilant when expanding the intervertebral space with the distractor system. The alignment of TMC was also found to affect the subsidence in the present study. It is obvious that the pressure on the adjacent endplate increased by an oblique TMC because of the sharp edge of the graft, which reduced the contact area (Additional file 1). The alignment of implant was a factor that should be concerned to be more in line with the anatomical characteristics of the cervical spine, especially the endplates. It is widely accepted that osteoporosis significantly associated with the increased risks for subsidence of implant after spinal surgery. Although we did not include the BMD owing to the high cost of Dual-energy X-ray absorptiometry (DEXA), HU values have been reported to be closely associated with bone quality and compressive capacity [12, 13]. In our study, the global cervical HU value was significantly lower in subsidence group than the non-subsidence group and the multivariate logistic regression analysis revealed that the poor bone density was the independent risk factor for subsidence of TMC. With an aging population, spinal surgeons must be aware of the importance of anti-osteoporosis treatments during the perioperative period and long-term follow-up.

The correlation between subsidence and clinical outcomes remains controversial. Some previous studies [2, 7, 24] reported that subsidence was responsible for poor clinical outcomes due to the loss of intervertebral height and recompression of the spinal cord and nerve root. However, subsidence of TMC did not usually appear to have a negative impact on successful clinical outcomes after surgery. Jang et al. [18] found that despite the prominent subsidence, SA and CSA increased at the final follow-up suggesting that subsidence may contribute to cervical lordosis. In this study, there were no significant differences in VAS and JOA scores immediately after surgery and at the final follow-up in the subsidence group which supported this view as well. However, excessive subsidence into the adjacent vertebra may result to catastrophic failure of the screw-plate system. Daubs et al. [11] also reported that 7 patients (30%) had early failures with severe subsidence and distal plate extrusion. Thus, to improve clinical outcomes, more effective measures for preventing excessive subsidence are needed. In this study, 3 patients suffered from screw breakage or loosening but subsidence is not the only cause of the reconstruction failure. Figure 4 shows a typical case of a 43-year-old man who experienced screw breakage in the early postoperative period while serious subsidence was not observed in the lateral radiograph. We believed that poor bone fusion rather than subsidence is the main cause of the reconstruction failure in this case. The incomplete fusion of bone grafts might not resist the stress caused by spinal movement, resulting in fatigue of screw-plate system. Dysphagia is a common complication of anterior cervical surgery. Its incidence varies, ranging from 1 to 79%, depending on the definition adopted [25]. Many factors are associated with the development of postoperative dysphagia, including soft tissue swelling, pharyngeal plexus denervation and scar tissue formation [25–27]. Dysphagia occurred in 3 patients (4.1%) in this study, and this low incidence rate may be ascribed to the single-level fusion, preoperative tracheal traction exercise and careful intraoperative soft-tissue handling. In all 3 cases, dysphagia was transient and spontaneously resolved without treatment.

There are a series of limitations that need to be addressed regarding this study. First, the main limitation lies in the retrospective nature. Second, it was a single-center study and the number of the cases was limited resulting to being underpowered to find more independent risk factors. Finally, the minimum follow-up of this study was 24 months and therefore the present findings cannot be interpreted as long-term results. Due to these limitations, prospective studies with large samples and long follow-up period are needed to further examine the results in our study.

## Conclusion

Our findings suggest that the value of RAE more than 1.18, alignment of TMC and poor bone mineral density are the risk factors for subsidence. TMC subsidence does not negatively affect the clinical outcomes after operation. Avoiding over expansion of intervertebral height, optimizing placing of TMC and initiation of anti-osteoporosis treatments 6 months prior to surgery might help surgeons to reduce subsidence after ACCF.

## Supplementary information

**Additional file 1. Figure S1.** The contact area between the TMC and endplate would be reduced when the cage was placed obliquely into the intervertebral space.

## Data Availability

The data are not publicly available due the privacy of patients included but are available from the corresponding author on reasonable request for academic research purpose.

## Abbreviations

ACCF: Anterior Cervical Corpectomy and Fusion
AUC: Area under the curve
BMD: Bone mineral density
CI: Confidence interval
CSA: Cervical sagittal angle
DEXA: Dual-energy X-ray absorptiometry scans
ES: Extent of subsidence
HAE: Height of anterior endplate
HPE: Height of posterior endplate
HU: Hounsfield Units
JOA: Japanese Orthopedic Association
OPLL: Ossified posterior longitudinal ligament
OR: Odds ratio
PACS: Picture archiving and communication system
RAE: Ratio of anterior endplate
ROC: Receiver operating characteristics (ROC) curve
RPE: Ratio of posterior endplate
SA: Segmental angle
TMC: Titanium mesh cage
VAS: Visual Analog Scale
