# Supplementary material for: Risk factors for subsidence of titanium mesh cage following single-level anterior cervical corpectomy and fusion
Source: BMC Musculoskelet Disord. 2020 Jan 14;21:32. doi: 10.1186/s12891-019-3036-8 (PMC6961320; doi:10.1186/s12891-019-3036-8)
Supplement: Supplementary file 1 — Additional file 1. Figure S1. The contact area between the TMC and endplate would be reduced when the cage was placed obliquely into the intervertebral space. [file 12891_2019_3036_MOESM1_ESM.docx]

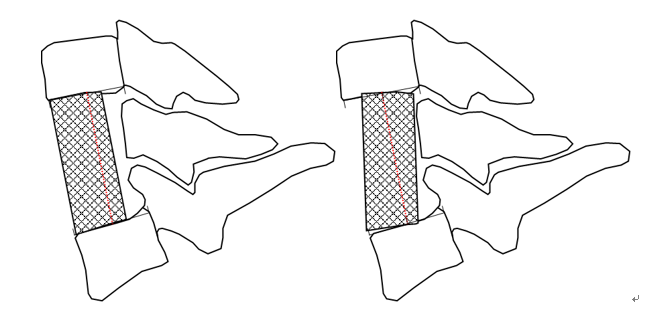


**Supplementary Figure** The contact area between the TMC and endplate would be reduced when the cage was placed obliquely into the intervertebral space.
